# Facing the inevitable: Rethinking medical education for an ageing population

**DOI:** 10.1016/j.fhj.2026.100558

**Published:** 2026-08-30

**Authors:** C.N. Uwakwem, N. Chance

**Affiliations:** North Central and East London Deanery, Barking, Havering and Redbridge University Hospitals NHS Trust, Romford, Essex RM7 0AG, United Kingdom

**Keywords:** Internal medicine training, National Health Service, Geriatrics

## Abstract

As the NHS faces increasing pressure from an ageing population and rising multimorbidity, Internal Medicine Trainees (IMTs) are required to navigate uncertainty, frailty, and end-of-life decision-making. For many, a geriatric rotation provides the first sustained exposure to patients for whom cure is unrealistic and goals of care are complex. This opinion piece examines how geriatric training reshapes trainee attitudes towards investigation, escalation, and death. While IMT training often reinforces investigation-driven practice, geriatric medicine foregrounds frailty, predictable illness trajectories, and anticipatory decision-making. Exposure to Universal Care Planning, Treatment Escalation Plans, and senior-led conversations about uncertainty legitimises non-intervention as an active clinical decision. Strengthening education around frailty, uncertainty, and death during IMT training may reduce non-beneficial investigation, improve patient-centred care, and help alleviate avoidable pressures on an increasingly constrained NHS.

## Learning to let go: what geriatric rotations teach internal medicine trainees about death, investigation, and the future of the NHS

For many internal medicine trainees (IMTs), a rotation in geriatric medicine represents the first sustained encounter with patients for whom recovery is uncertain and cure is often unrealistic. Trained within a system that rewards diagnostic thoroughness and escalation, trainees may arrive on geriatric wards equipped with algorithms but unprepared for ambiguity. Yet as the NHS faces increasing pressure from an ageing population, the lessons learnt during these rotations may be among the most important of modern medical training.

## The investigation reflex in training

Internal medicine training fosters a culture in which action is equated with safety. Investigations are often the visible marker of diligence, particularly in acute care. On geriatric wards, however, trainees quickly observe that investigations frequently do not alter management, but uncover incidental findings, or prolong admissions without improving outcomes. This is especially true in patients living with frailty, where physiological reserve is limited and illness trajectories are often predictable.^1^

Despite this, trainees may feel uneasy withholding tests. In the absence of clearly documented uncertainty care plans or ceilings of care, continued investigation can feel safer than restraint. This reflects not ignorance, but a training environment that has not consistently legitimised proportionate decision-making.^2^

## Frailty, trajectories, and realistic medicine

Geriatric medicine exposes trainees to a fundamentally different clinical lens. Frailty, now recognised as a measurable and prognostically powerful construct, reframes illness as cumulative vulnerability rather than isolated pathology. Observational studies in older adults with frailty suggest that decline towards the end of life is often gradual or stepwise rather than abruptly reversible, reinforcing the importance of anticipatory rather than reactive decision-making.^1^

Understanding these patterns allows clinicians to anticipate outcomes and plan care accordingly. For trainees, this represents a shift from reactive investigation to anticipatory thinking – a skill rarely taught explicitly elsewhere in the IMT programme.

## Universal care planning: from concept to practice

Universal Care Planning (UCP) is increasingly recognised as central to high-quality care for people with unpredictable illness trajectories.^3^ When implemented early and revisited regularly, UCP enables clinicians to align investigations and treatments with patient priorities, reducing unwanted escalation and distress.

In practice, however, internal medicine trainees often encounter UCPs late or not at all. Discussions about prognosis and preferences may be deferred until crisis points, at which stage investigations become default actions rather than considered choices. Evidence suggests that early advance care planning improves alignment between care delivered and patient values, while reducing non-beneficial interventions.^4^

Geriatric rotations provide a rare opportunity to observe senior clinicians conducting these conversations well – framing uncertainty honestly, acknowledging limits, and documenting decisions clearly. These rotations also reveal the challenge of ensuring that conversations about goals of care are translated into clear, consistent clinical decisions through tools like Treatment Escalation Plans (TEPs) and DNACPR orders.

Evidence suggests that current approaches to Treatment Escalation Plans and DNACPR decisions remain highly variable.^5^ There remains insufficiently clear guidance about which level of clinician is expected to initiate these conversations, and whether patients and their families should simply be made aware of decisions or play an active role in decision-making – ambiguity that is reflected in inconsistent practice between clinicians. Legal analyses of DNACPR decision-making highlight how this lack of clarity can heighten anxiety about potential ethical or legal consequences, inadvertently discouraging open discussion rather than supporting it.^6^

For internal medical trainees, this variability exposes a gap in formal education: training in TEP and DNACPR decision-making is often implicit, reliant on observation rather than structured guidance. With clearer training, IMTs can develop confidence in these decisions and, in turn, provide leadership and consistency for more junior colleagues.

## Learning to talk about death

Perhaps the most profound educational gap exposed during geriatric placements is discomfort with death itself. Many trainees report minimal formal teaching on how to discuss dying, prognosis, or uncertainty. As a result, investigations may be serving as a substitute for conversation – a way of deferring difficult discussions rather than confronting them.^7^

Experienced geriatricians often model a different approach, reframing death not as medical failure but as an expected outcome of ageing and illness. This reframing is not nihilistic; rather, it enables care to be redirected towards comfort, dignity, and patient-defined goals.^8^ For trainees, witnessing this approach can legitimise conversations that otherwise feel difficult or are avoided.

## At what price?

The implications of these behaviours extend beyond individual patients. Projections suggest a substantial rise in the number of people who will require palliative and end-of-life care over the coming decades, driven largely by population ageing and multimorbidity.^9^ Without cultural change, the NHS risks increasing use of investigations and hospital admissions that neither improve outcomes nor reflect patient preferences.

With care that is guided by patients’ priorities and goals, we can reduce the healthcare burden and improve patient experience among older adults with multiple chronic conditions (1). For trainees, learning these principles early may be as important for system sustainability.

The consequences of how we investigate and escalate care in older adults are not only clinical; they shape the flow of wards, the workload of staff, and the financial sustainability of the NHS. Evidence suggests that when dying is not recognised and goals of care are not discussed early, investigation-driven care can generate substantial costs with limited patient benefit. A recent retrospective study of patients aged 80 years and over demonstrated that, in the final six months of life, a single NHS hospital performed 699 radiological procedures in 96 patients, with the cost of CT and MRI imaging alone estimated at approximately £312,000 annually.^10^

These figures represent more than financial expenditure; they reflect the bed-days occupied by patients awaiting further tests, the prolonged admissions, and the downstream pressures on staffing and patient flow. How often, as trainees, do we see frail patients lingering on the wards for days awaiting investigations that might have been avoided with earlier recognition of their frailty and appropriate planning? For trainees, these patterns are often normalised, reinforcing the belief that continued investigation is the safest course.

Yet re-orientating medical education towards earlier recognition of frailty – which in turn allows proportionate investigation, and confident escalation planning – could reduce avoidable testing, shorten admissions, and ease workforce strain. In this way, learning when to let go is not only a clinical skill, but a vital response to the growing mismatch between NHS capacity and population need.

## Re-imagining IMT education

If investigation-driven care contributes to avoidable system strain, then medical education must be part of the solution. Geriatric rotations should not be treated merely as service posts but recognised as foundational placements in which trainees learn to navigate uncertainty, limits, and mortality. These rotations expose trainees to clinical realities that are increasingly common across all specialties: frailty, multimorbidity, and the need to balance intervention with restraint.

Embedding explicit teaching on frailty, uncertainty care planning, and proportionate investigation – supported by senior role-modelling – could have lasting impact on how doctors practise beyond geriatrics.^2^ Improving education about death is not about reducing care but about refining it. By legitimising uncertainty, prioritising conversation over investigation, and reframing death as part of care rather than its failure, trainees can develop the confidence to make decisions that are both clinically sound and ethically grounded.

For internal medicine trainees, geriatric medicine offers an education in what medicine cannot fix – and, crucially, what it still can offer. Learning when not to investigate is not inaction; it is clinical maturity. In a health system facing increasing demand and finite resources, this maturity is essential to the future of the NHS.

## CRediT authorship contribution statement

**C.N. Uwakwem:** Writing – review & editing, Writing – original draft, Methodology, Conceptualization. **N. Chance:** Writing – review & editing, Conceptualization.

## Funding

This article did not receive any specific grant from funding agencies in the public, commercial, or not-for-profit sectors.

## Declaration of competing interest

The authors declare that they have no known competing financial interests or personal relationships that could have appeared to influence the work reported in this paper.

## References

[1] Tinetti M.E., Esterson J., Ferris R., Posner P., Blaum C.S. (2022). Patient priority–directed decision making and care for older adults with multiple chronic conditions. Clin Geriatr Med.

[2] Oliver D. (2021). Let’s stop measuring what doesn’t matter in the care of older people. Future Healthc J.

[3] Royal College of Physicians (2021). Uncertainty Care Planning: Supporting People During Unpredictable Illness.

[4] Rietjens J.A.C., Sudore R.L., Connolly M. (2017). Definition and recommendations for advance care planning: an international consensus supported by the European Association for Palliative Care. Lancet Oncol.

[5] Warner B.E., Lound A., Grailey K., Vindrola-Padros C., Wells M., Brett S.J. (2023). Perspectives of healthcare professionals and older patients on shared decision-making for treatment escalation planning in the acute hospital setting: a systematic review and qualitative thematic synthesis. eClinicalMedicine.

[6] Bows H., Herring J. (2022). DNACPR decisions during COVID-19: an empirical and analytical study. Med Law Rev.

[7] Smith A.K., White D.B., Arnold R.M. (2013). Uncertainty—the other side of prognosis. N Engl J Med.

[8] Smith A.K., Sudore R.L., Perez-Stable E.J. (2022). Palliative care for patients with serious illness: addressing the gap between evidence and practice. N Engl J Med.

[9] Etkind S.N., Bone A.E., Gomes B. (2020). How many people will need palliative care in the future? Projections for England and Wales to 2040. Lancet Public Health.

[10] Pattnaik S., Fereday L., O'Callaghan R. (2023). Investigations and interventions in the last six months of life in patients aged 80 years and over: a retrospective study in a UK hospital. Age Ageing.

